# Good recovery is not full recovery: emotion recognition deficits and different regulation strategies in mild right-hemisphere ischemic stroke survivors

**DOI:** 10.3389/fpsyg.2026.1803288

**Published:** 2026-04-09

**Authors:** Álvaro Ruiz-García, Beatriz García-Rodríguez

**Affiliations:** 1 Universidad Nacional de Educación a Distancia, Basic Psychology II, Madrid, Spain; 2 Hospital Universitario 12 de Octubre, Madrid, Spain; 3 Faculty of Psychology and Health Sciences, Distance University of Madrid, Madrid, Spain

**Keywords:** emotion, emotion identification, emotion regulation, ischemic stroke, right-hemisphere

## Abstract

**Introduction:**

Mild right-hemisphere ischemic stroke can affect socioemotional functioning even in patients with excellent functional recovery, yet facial emotion identification and habitual emotion regulation remain undercharacterized in this population. This study examined both domains in stroke survivors with little-to-no residual disability compared to healthy controls.

**Methods:**

Fifty-three mild right-hemisphere ischemic stroke survivors (modified Rankin Scale < 2; chronic phase, 12–24 months post-stroke) and 60 neurologically healthy controls completed a 24-item forced-choice identification task covering six basic emotions and the Emotion Regulation Questionnaire (ERQ). Group differences and the associations between regulation and recognition were examined with repeated-measures ANOVA and ANCOVA, including general cognitive status (MoCA) and ERQ indices as covariates.

**Results:**

In the unadjusted model, stroke participants showed lower overall accuracy and a significant Emotion × Group interaction, with the clearest group differences for anger, disgust, and fear. The stroke group also reported markedly higher expressive suppression and lower cognitive reappraisal. When MoCA was included as the sole covariate, the between-subjects group effect on overall accuracy was no longer significant, indicating that the global accuracy decrement is substantially tied to the mild cognitive sequelae of right-hemisphere stroke; however, the Emotion × Group interaction retained significance with an identical effect size, becoming non-significant only in the extended model that simultaneously controlled for both MoCA and ERQ indices. Expressive suppression showed a significant emotion-dependent association with recognition accuracy after adjustment for both ERQ and MoCA, whereas reappraisal did not. Formal ANCOVAs confirmed that regulation differences were not accounted for by cognitive status.

**Discussion:**

These findings reveal a functional dissociation: the overall level of facial emotion identification is sensitive to general cognitive integrity, whereas habitual emotion regulation represents a higher-order, cognitively independent dimension of socioemotional functioning that is robustly and independently affected by mild stroke. The results argue for dedicated socioemotional assessment — and particularly regulation-focused characterization — in well-recovered stroke survivors, and motivate future work linking these profiles to lesion mechanisms and targeted interventions.

## 1 Introduction

Emotions are central to human adaptation. They organize perception, guide action selection, and scaffold social interaction (Ekman, 1992). A cornerstone of this is emotion perception, because it supports rapid inferences about other people’s internal states and action tendencies, enabling adaptive interpersonal behavior (Adolphs, 2002). A substantial portion of this work has focused on recognition of basic emotions from nonverbal cues (especially faces), supported by relatively well-described distributed neural systems that include occipitotemporal visual cortices, the amygdala, orbitofrontal regions, and frontoparietal control networks (Adolphs, 2002; Ekman, 1992). At the same time, there is increasing recognition that emotional meaning is not “read off” from perceptual cues alone: contextual information and learned conceptual knowledge can shape how signals are interpreted, blurring strict boundaries between basic and more complex emotional states and highlighting the need for models that integrate bottom-up and top-down contributions (Barrett, 2022).

Within clinical neuropsychology, stroke provides a particularly informative model to examine how focal vascular lesions perturb emotion processing, although, in stroke research, emotional sequelae have traditionally been discussed in terms of mood symptoms (e.g., post-stroke depression is among the most prevalent neuropsychiatric consequences of stroke, affecting approximately one third of survivors; Robinson and Jorge, 2016). Meta-analytic evidence indicates that social cognitive difficulties are common after stroke, with moderate-to-large impairments reported across domains such as social perception, theory of mind, and social behavior (Adams et al., 2019). Yet, in routine care, emotion-recognition problems are often under-assessed because they may not be spontaneously reported and can be easily missed when standard cognitive screening is normal (Aben et al., 2020). In a large ischemic stroke cohort, impaired emotion recognition was observed in a substantial subset of patients, and importantly, deficits were still present in individuals without overt cognitive disorder in traditional domains (Aben et al., 2020). Taken together, these findings underscore that emotion identification is both clinically relevant and potentially independent of global cognitive status, with downstream implications for social functioning and quality of life (Aben et al., 2020).

A central theoretical question is whether particular hemispheric or network-level mechanisms are disproportionately implicated in these deficits. Classic accounts of emotional laterality have proposed that the right hemisphere plays a privileged role in processing nonverbal emotional communication, especially when meaning must be derived from facial or prosodic cues rather than explicit situational semantics (Blonder et al., 1991). More recent syntheses situate such proposals alongside alternative frameworks, including valence-based and approach–withdrawal models, acknowledging that laterality effects can depend on stimulus modality, task demands, and the specific affective computations required (Davidson, 1992; Gainotti, 2024). Lesion-based evidence further suggests that deficits in recognizing certain emotions may be associated with damage to specific right-hemisphere structures (e.g., amygdala/insula involvement for fear/disgust-related processing), consistent with the idea that emotion recognition relies on partially specialized but interacting nodes within a broader network (Adolphs, 2002; Tippett et al., 2018).

Critically, however, emotion recognition after stroke is unlikely to be explained by lesion location alone. Individual differences in how people regulate and interpret emotions may contribute to variability in performance, and this may be especially important in relatively mild stroke presentations where gross functional outcomes appear favorable. Emotion regulation refers to the processes by which individuals influence which emotions they have, when they have them, and how they experience and express them (Gross, 1998). Within Gross’s (1998) process model, regulation can occur at multiple stages. Cognitive reappraisal involves reinterpreting the meaning of an emotionally significant situation in order to alter its emotional impact —for example, viewing a medical diagnosis as a manageable challenge rather than a catastrophe— and is classified as an antecedent-focused strategy because it intervenes before the full emotional response has been generated (Gross, 1998; Gross and John, 2003). Expressive suppression, by contrast, involves inhibiting the outward behavioral expression of an ongoing emotion —for example, masking distress with a neutral facial expression— and is classified as a response-focused strategy because it operates after the emotion has already been generated (Gross, 1998; Gross and John, 2003). These strategies show robust associations with affective and interpersonal outcomes in healthy samples, with reappraisal generally linked to more adaptive profiles (e.g., greater positive affect, better social relationships, lower psychological symptoms) than suppression, which tends to be associated with higher negative affect, reduced emotional expressiveness, and interpersonal difficulties (Gross and John, 2003). Mechanistically, reappraisal draws on cognitive control systems that can modulate emotion-relevant perceptual and appraisal processes, implicating frontoparietal and prefrontal contributions to emotion-related behavior (Ochsner and Gross, 2005).

Despite this strong theoretical and empirical foundation, comparatively little work has examined emotion regulation tendencies in stroke cohorts, and even fewer studies have tested whether habitual regulation styles help explain variability in emotion identification performance. The available evidence suggests that emotion regulation is clinically meaningful after stroke and relates to broader social functioning (Cooper et al., 2015). In a study of community-dwelling stroke survivors, Cooper et al. (2015) found that higher expressive suppression was associated with reduced social participation, while greater cognitive reappraisal predicted more favorable social engagement outcomes, even after controlling for functional disability and depressive symptoms. These findings suggest that habitual regulation style shapes not only affective outcomes but also behavioral participation in everyday social contexts, which in turn may be directly influenced by the capacity to accurately read emotional signals in others. Indeed, the experience of one’s own emotions and the recognition of emotions in others are not independent processes: habitual regulation strategies modulate the generation, differentiation, and outward expression of internal affective states, and these same processes share overlapping neural substrates with those involved in decoding facial expressions (Ochsner and Gross, 2005). Individuals who chronically inhibit expressive emotional output may develop attenuated sensitivity to external affective signals, potentially impairing the recognition of others’ emotional expressions (Gross and John, 2003; Ochsner and Gross, 2005) —a mechanism that is particularly relevant in a population where right-hemisphere lesions may already compromise the neural resources supporting socioemotional perception. Despite this, the field lacks a systematic integration of regulation constructs with experimental measures of emotion perception, particularly in well-recovered patients. This gap is notable because a person’s typical regulatory stance could plausibly influence how they allocate attention to emotional cues, how flexibly they interpret ambiguous expressions, and how readily they use contextual information to disambiguate affective meaning—mechanisms that are directly relevant to both basic emotion identification and the construction of more complex emotional understanding (Barrett, 2022; Ochsner and Gross, 2005).

Against this backdrop, the present study focuses on survivors of mild right-hemisphere ischemic stroke with excellent functional recovery, operationalized as modified Rankin Scale (mRS) scores < 2, reflecting little-to-no residual disability in daily life (Rankin, 1957; van Swieten et al., 1988). Studying this subgroup is theoretically and clinically informative: if subtle deficits in emotion identification and their modulation by regulation style can be detected even when global function is preserved, such findings would argue for including socio-emotional outcomes in routine post-stroke characterization and would help refine models of emotional laterality by separating “functional recovery” from “social-cognitive recovery.”

We therefore examine (a) whether individuals with mild right-hemisphere ischemic stroke differ from demographically comparable controls in global accuracy and emotion-specific patterns of facial emotion identification, and (b) whether individual differences in habitual emotion regulation (cognitive reappraisal and expressive suppression) are associated with emotion identification performance and/or account for variance beyond group membership. Based on the theoretical and empirical considerations outlined above, we formulate the following *a priori* hypotheses. First, regarding emotion recognition, we predict that the stroke group will show selectively reduced accuracy for emotions whose recognition depends preferentially on right-hemisphere structures implicated in threat- and aversion-related processing (particularly anger and disgust), rather than a uniform decrement across all emotion categories, consistent with lesion-based evidence linking right insula and amygdala pathology to specific recognition deficits (Adolphs, 2002; Tippett et al., 2018). Second, regarding emotion regulation, we predict that the stroke group will report significantly higher expressive suppression and lower cognitive reappraisal compared to controls, consistent with the evidence that right-hemisphere stroke affects socioemotional management (Cooper et al., 2015) and that the neural substrates of reappraisal include prefrontal and frontoparietal regions that are vulnerable to vascular lesions (Ochsner and Gross, 2005). Third, as an exploratory prediction, we expect that individual differences in habitual regulation style —particularly expressive suppression— will account for variance in emotion recognition performance over and above group membership, given the mechanistic links between regulatory and perceptual processes outlined above.

## 2 Materials and methods

### 2.1 Participants

A total of 113 individuals participated in the study: 53 stroke survivors (30 men, 56.6%, mean age = 52.25, SD = 8.58) with mild right-hemisphere ischemic stroke and excellent functional recovery (modified Rankin Scale, mRS < 2), all evaluated in the chronic phase of recovery (12–24 months post-stroke), and 60 neurologically healthy controls (37 men, 61.7%, mean age = 49.80, SD = 8.35). Full demographic and clinical characteristics are reported in Table 1. The two groups were broadly comparable across all variables; the only significant between-group difference was observed for the MoCA (Nasreddine et al., 2005), with stroke survivors scoring lower than controls (M = 27.15 vs. 28.53), although both groups performed within the non-impaired range (see Table 1).

**Table 1:** Sociodemographic and clinical variables.

Variable	Stroke patients (M, SD)	Control group (M, SD)	Test statistic	*p*
*N*	53	60		
Sex, *n* (%)			χ^2^(1) = 0.299	0.585
Men	30 (56.6)	37 (61.7)		
Women	23 (43.4)	23 (38.3)		
Age (years)	52.25 (8.58)	49.80 (8.35)	t(108.5) = 1.534	0.128
Years of education	13.64 (2.59)	13.67 (2.72)	t(110.4) = −0.060	0.952
MoCA	27.15 (1.76)	28.53 (1.84)	t(111) = −4.074	<0.001
BDI	5.89 (4.34)	5.05 (2.09)	t(111) = 1.269	0.206

MoCA, Montreal Cognitive Assessment; BDI, Beck Depression Inventory.

Exclusion criteria for both groups included moderate-to-severe depressive symptomatology (BDI > 15), clinically significant cognitive impairment on screening (MoCA < 26), prior diagnoses of psychiatric or neurological disorders (other than stroke in the patient group), and current use of psychotropic medication. All participants provided written informed consent prior to participation, and study procedures adhered to the ethical standards of the Declaration of Helsinki. Participants were not financially compensated for their participation.

### 2.2 Instruments

#### 2.2.1 Emotional facial expression identification (EFE) task

Facial emotion recognition was assessed with a forced-choice identification paradigm comprising 24 color, computer-generated facial expressions representing six basic emotions (happiness, sadness, surprise, fear, anger, and disgust), with four exemplars per emotion. The stimuli were created via 3D facial modeling software (Poser 6; Curious Labs, Santa Cruz, CA) and were parametrically built from the muscle movements specified in the Facial Action Coding System (FACS; see Table 2), ensuring that each expression reflected a standardized configuration of action units (AUs) rather than idiosyncratic features of a photographed actor (Ekman and Friesen, 1978; Ekman et al., 2002). The facial configurations followed canonical AU patterns for each emotion: happiness (AU 6 + 12 + 26), sadness (AU 1 + 4 + 15), surprise (AU 1 + 2 + 5 + 26), fear (AU 1 + 4 + 5 + 20 + 26), anger (AU 4 + 5 + 24), and disgust (AU 4 + 9 + 10 + 15) (Ekman and Friesen, 1978; Ekman et al., 2002). Stimulus development and AU implementation were conducted by an experienced FACS coder (Johannes Heiner Ellgring), which is consistent with the methodological approach used in prior work employing the same stimulus family and task logic to study attentional interference effects on emotion identification across clinical and aging samples (García-Rodríguez et al., 2009, 2012a, 2012b).

**Table 2:** Description of facial action units (FACS)*.

Emotion	Facial action units
Happiness	6 + 12 + 26
Sadness	1 + 4 + 15
Surprise	1 + 2 + 5 + 26
Fear	1 + 4 + 5 + 20 + 26
Anger	4 + 5 + 24
Disgust	4 + 9 + 10 + 15

*1 = medial portion of the frontalis muscle (internal levator of the eyebrow), 2 = lateral portion of the frontalis muscle (external levator of the eyebrow), 4 = corrugator supercilii + depressor supercilii + procerus muscle (lowering/frowning of the eyebrow), 5 = levator palpebrae superioris muscle (elevating the upper eyelid), 6 = orbital portion of the orbicularis oculi muscle (elevating the cheek, crow’s feet), 9 = levator labii superioris alaeque nasi muscle (wrinkling of the nose), 10 = levator labii superioris muscle (elevating the upper lip), 12 = zygomaticus major muscle (pulling the corner of the mouth upwards), 15 = depressor anguli oris muscle (lowering the corner of the mouth), 20 = risorius muscle (lateral stretching of the lip, forced smile), 24 = marginal portion of the orbicularis oris muscle (pressing the lips together), 26 = relaxation of the jaw elevator muscles (masseter, temporalis, medial pterygoid) → passive lowering of the jaw.

**Figure 1:**
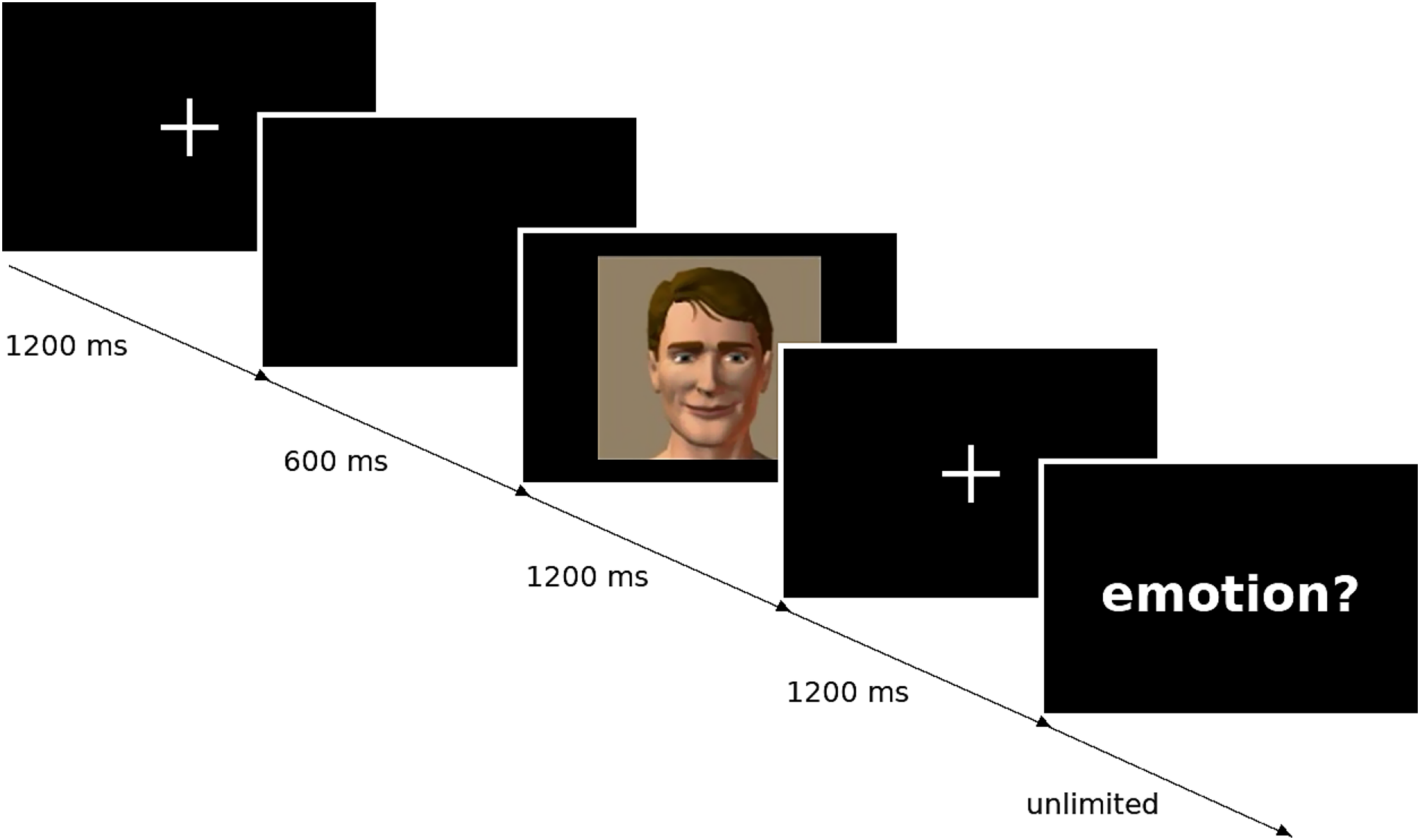
The figure depicts a representative trial sequence: fixation cross (1,200 ms), blank screen (600 ms), facial stimulus (1,200 ms), and final fixation cross (1,200 ms). The final panel represents the response prompt; in the actual task, participants selected from the six labeled response options (happiness, sadness, surprise, fear, anger, disgust) presented on screen. The full response interface is not reproduced within the figure panel to preserve legibility of the trial sequence.

Each trial began with a centrally presented fixation cross (1,200 ms; see Figure 1), followed by a blank black screen (600 ms), then an emotional face stimulus (1,200 ms), and a final fixation cross (1,200 ms). After offset of the final fixation cross, the response phase began: the six emotion labels (happiness, sadness, surprise, fear, anger, disgust) were displayed on the screen and remained visible until the participant indicated their choice, with response time unlimited to minimize speed–accuracy tradeoffs and to keep the dependent variable focused on identification accuracy rather than motor speed. This ensured that participants had access to the response options throughout the decision process, reducing demands on verbal memory for emotion category names. The presentation order was randomized to mitigate sequence learning and familiarity effects, and no performance feedback was provided during or after the task.

Participants indicated their responses verbally or by pointing, and the experimenter recorded responses manually (rather than via keyboard/button press), a deliberate choice to reduce potential confounding by visuomotor slowing, coordination constraints, or technical artifacts —particularly relevant when testing older adults or neurological patients (García-Rodríguez et al., 2009, 2012a, 2012b)— so that the recorded outcome more directly indexed emotion identification ability. Per-emotion accuracy (range 0–4 per category) served as the outcome measure for all analyses.

A key advantage of using standardized, computer-generated expressions is experimental control: intensity, viewpoint, and facial movement patterns can be held constant across exemplars, improving internal validity and comparability across participants (García-Rodríguez et al., 2009, 2012a, 2012b). In addition, converging evidence indicates that avatar-based emotional expressions can engage core affective circuitry in a manner broadly comparable to human facial stimuli; for example, event-related fMRI studies have shown reliable amygdala activation to both human and avatar emotional expressions, supporting the utility of virtual faces for neurocognitive investigations (Moser et al., 2007).

The task was programmed with Python and administered using PsychoPy (Peirce et al., 2019), an open-source software environment for the design and execution of neuroscience and psychology experiments. Stimuli were displayed on a 19-inch monitor at a standard viewing distance of approximately 60 cm.

#### 2.2.2 Global cognitive screening

Global cognitive status was assessed with the Montreal Cognitive Assessment (MoCA), a brief 30-point screening tool covering multiple domains (e.g., attention, executive functions, memory, language, visuospatial abilities, and orientation). The MoCA was used to characterize cognitive status and to support exclusion of participants with clinically relevant global impairment (cutoff score < 26; Nasreddine et al., 2005).

#### 2.2.3 Emotion regulation

Habitual emotion regulation strategies were evaluated using the Emotion Regulation Questionnaire (ERQ; Gross and John, 2003), a 10-item self-report measure that indexes two widely studied strategy families: cognitive reappraisal (6 items) and expressive suppression (4 items). Items are rated on a 7-point Likert scale (1 = strongly disagree; 7 = strongly agree) and averaged within subscales, yielding a mean score ranging from 1 to 7 for each strategy; higher scores reflect more frequent habitual use of the respective strategy. The ERQ was originally developed to capture stable individual differences in these processes and has been linked to affective experience, interpersonal functioning, and social behavior (Gross and John, 2003). A validated Spanish adaptation is available and has shown adequate psychometric performance in Spanish-speaking samples (Cabello et al., 2013). In the present study, ERQ reappraisal (ERQ_Cog) and ERQ suppression (ERQ_Sup) were treated as individual-difference predictors and/or covariates in models examining emotion identification outcomes.

#### 2.2.4 Depressive symptoms

Depressive symptom severity was assessed using the Beck Depression Inventory–II (BDI-II), a 21-item self-report inventory with responses scored on a 0–3 scale, producing a total score typically ranging from 0 to 63 (Beck et al., 1996). The BDI-II was used to quantify depressive symptom load and, consistent with the study’s exclusion criteria, to identify and exclude individuals with clinically significant depressive symptomatology. Participants scoring above 15 on the BDI-II were excluded, in accordance with the threshold for moderate-to-severe symptomatology established in the Spanish normative standardization (Sanz et al., 2003), which classifies scores of 16 and above as indicative of clinically meaningful depressive pathology. Spanish psychometric data for the BDI-II have been reported in large general-population samples, supporting its reliability and factorial validity in Spain (Sanz et al., 2003).

### 2.3 Procedure

Participants were evaluated individually in a single session of approximately 60 min. The evaluation sequence was as follows:

1. Cognitive assessment.
2. Depression assessment.
3. Emotional regulation questionnaires.
4. Emotional identification test.

The study received ethical approval from the Hospital 12 de Octubre Ethics Committee (Madrid, Spain). Stroke group participants were recruited during follow-up consultations at the Neurology Unit of Hospital Universitario 12 de Octubre and were evaluated individually at the hospital. Control participants were recruited as volunteers from the Madrid metropolitan area and were evaluated at various community locations in Madrid. All sessions were conducted individually by the same experimenter, following the standardized evaluation sequence described above. Potential differences in assessment setting between groups are acknowledged as a limitation of the design.

### 2.4 Data analyses

Group differences in sample characteristics were examined using *χ*^2^ tests for categorical variables (sex) and independent-samples *t* tests for continuous variables (age, education, MoCA, BDI), applying Welch’s correction when Levene’s test indicated heteroscedasticity. Emotion identification accuracy was analyzed with a repeated-measures ANOVA (implemented within the General Linear Model [GLM] framework in SPSS) including Emotion (six levels: joy, sadness, anger, disgust, surprise, fear) as the within-subject factor and Group (stroke vs. control) as the between-subject factor. When sphericity was violated, Greenhouse–Geisser corrections were applied to univariate within-subject tests, and multivariate results were additionally reported using Pillai’s trace. Significant Emotion × Group effects were followed up with Bonferroni-adjusted simple comparisons of groups within each emotion. Group differences in emotion regulation were tested with independent-samples t tests for ERQ_Cog and ERQ_Sup, reporting Cohen’s d and 95% confidence intervals. To examine the integrative relationship between emotion regulation and identification performance, a repeated-measures ANCOVA re-estimated the Emotion × Group model including ERQ_Cog and ERQ_Sup as covariates; given evidence of unequal covariance matrices, Pillai’s trace was prioritized for multivariate inference, alongside Greenhouse–Geisser-corrected univariate tests. Additionally, to examine whether group differences in emotion identification could be accounted for by cognitive status independently of regulation, a supplementary repeated-measures ANCOVA was estimated including MoCA as the sole covariate alongside Group and Emotion. To formally evaluate whether group differences in habitual emotion regulation were independent of cognitive status, two univariate ANCOVAs were conducted with ERQ_Sup and ERQ_Cog as dependent variables, Group as the between-subject factor, and MoCA as the covariate. The Beck Depression Inventory (BDI) was administered as a screening instrument to exclude participants with moderate-to-severe depressive symptomatology (cut-off > 15) and was not included as a covariate in any analysis. This decision rests on two grounds. First, BDI scores did not differ significantly between groups (t(111) = 1.269, *p* = 0.206, d = 0.240), so BDI does not meet the standard criteria for a confounding variable and its inclusion would not serve a statistical control function. Second, habitual emotion regulation strategies —particularly expressive suppression and reduced cognitive reappraisal— are well-established antecedents and maintaining factors of depressive symptomatology, not merely correlates (Gross and John, 2003; Ochsner and Gross, 2005). Adjusting for BDI in models predicting ERQ-based outcomes would therefore risk removing the variance most theoretically meaningful for characterizing the post-stroke regulatory profile, constituting a form of overadjustment bias analogous to the concern described above with respect to MoCA. Data were analyzed in IBM SPSS Statistics, Version 28.0 (IBM Corp., Armonk, NY, USA). Statistical significance was set at *α* = 0.05, and effect sizes are reported as partial eta squared (ηp^2^) for ANOVA/ANCOVA effects. All figures were generated using Python 3 (Python Software Foundation, https://www.python.org), with the Matplotlib library (Hunter, 2007). Standard error calculations were performed using the NumPy library (Harris et al., 2020).

## 3 Results

### 3.1 Emotion identification performance

Sphericity was violated (Mauchly’s W = 0.246, χ^2^ (14) = 153.007, *p* < 0.001); therefore, univariate within-subject effects were interpreted using Greenhouse–Geisser corrections (εGG = 0.681), and multivariate tests (Pillai’s trace) were also reported.

There was a robust main effect of Emotion (Greenhouse–Geisser: *F*(3.403, 377.696) = 65.932, *p* < 0.001, ηp^2^ = 0.373; Pillai = 0.771, *F*(5, 107) = 72.150, *p* < 0.001, ηp^2^ = 0.771), indicating that accuracy differed substantially across emotions.

There was also a significant main effect of Group (*F*(1, 111) = 9.225, *p* = 0.003, ηp^2^ = 0.077), reflecting lower overall accuracy in the stroke group when averaged across emotions. Critically, the Emotion × Group interaction was significant [Greenhouse–Geisser: F(3.403, 377.696) = 2.622, *p* = 0.043, ηp^2^ = 0.023; Pillai = 0.131, F(5, 107) = 3.228, *p* = 0.009, ηp^2^ = 0.131], showing that the magnitude of the group difference depended on the specific emotion.

**Figure 2:**
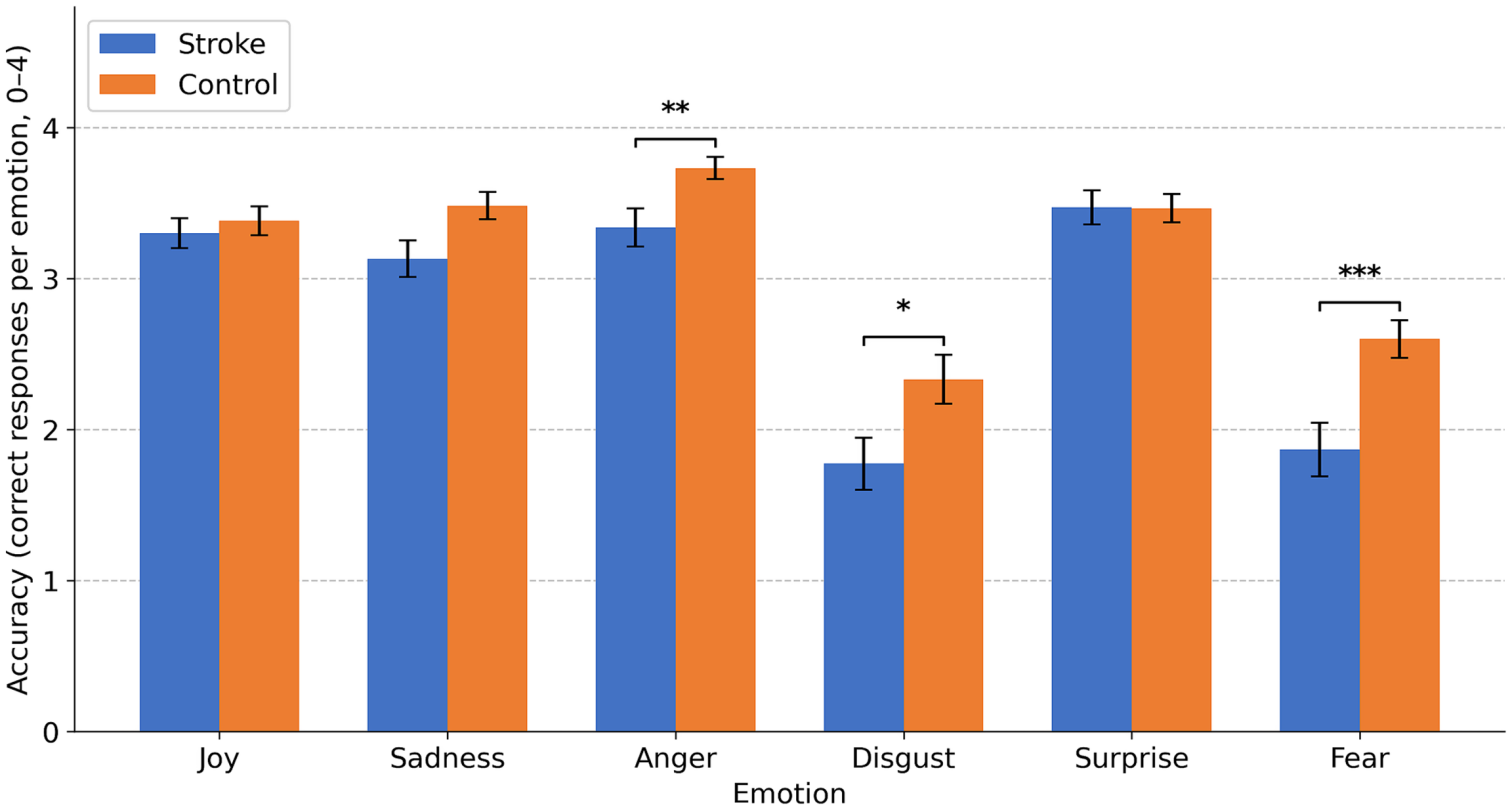
Bar plots show mean numbers of correct responses per emotion (out of 4) for the stroke group and the control group, with error bars depicting standard errors of the mean. Asterisks indicate significant Bonferroni-adjusted group differences: *p < 0.05; **p < 0.01; ***p < 0.001.

Bonferroni-adjusted comparisons (Group within each emotion; see Figure 2) indicated significantly lower performance in the stroke group for fear (stroke: M = 1.868, SD = 1.287; control: M = 2.600, SD = 0.960; *Δ* = −0.732, *p* < 0.001), disgust (stroke: M = 1.774, SD = 1.266; control: M = 2.333, SD = 1.258; Δ = −0.560, *p* = 0.020), and anger (stroke: M = 3.340, SD = 0.919; control: M = 3.733, SD = 0.578; Δ = −0.394, *p* = 0.007). Differences were not significant for joy (*p* = 0.557) or surprise (*p* = 0.834). Sadness showed a borderline effect after correction (Δ = −0.353, *p* = 0.053).

### 3.2 Group differences in emotion regulation

**Figure 3:**
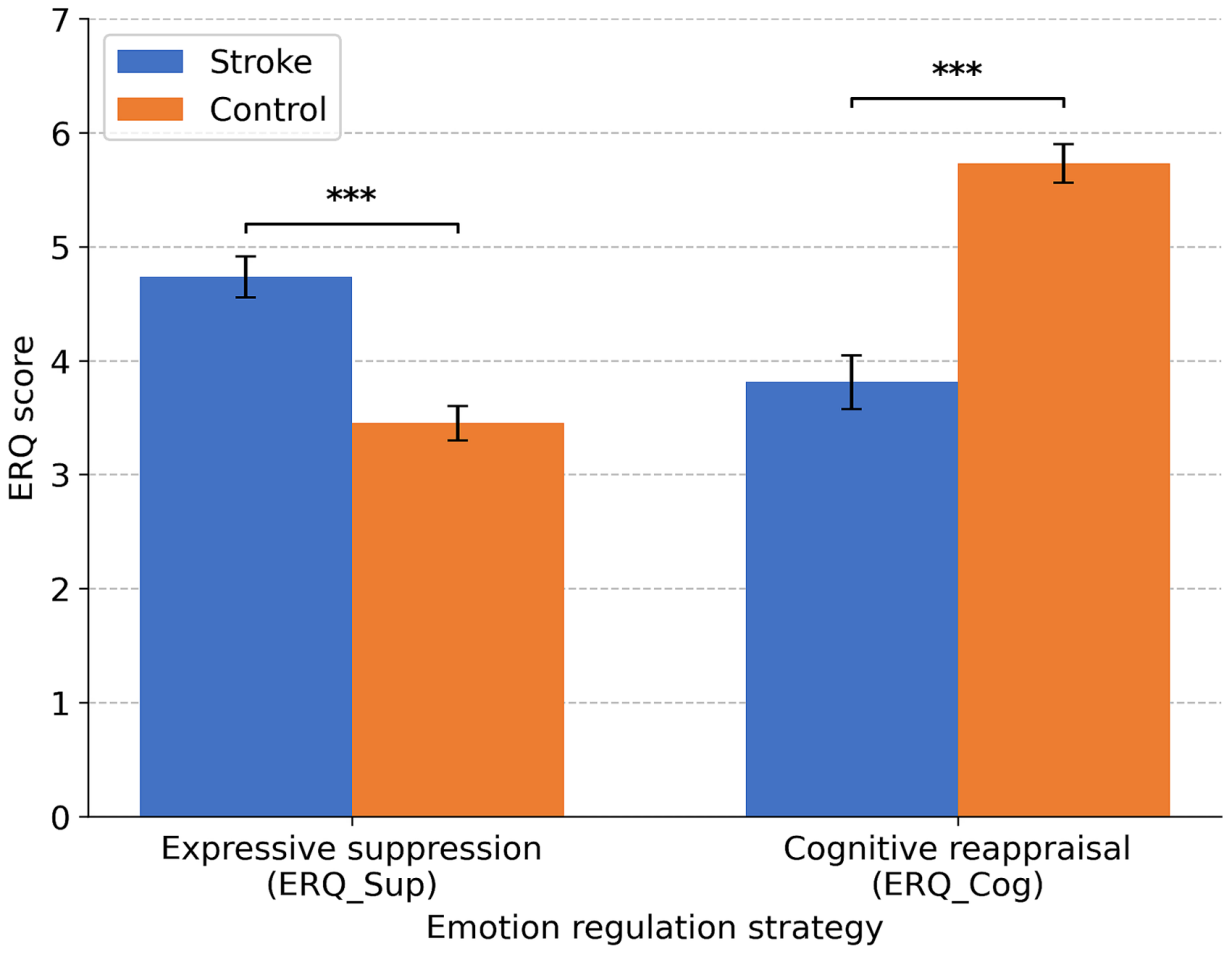
Bar plots show means of ERQ scores for the stroke group and the control group, with error bars depicting standard errors of the mean. Asterisks indicate significant Bonferroni-adjusted group differences: ***p < 0.001.

The stroke group reported markedly higher expressive suppression (ERQ_Sup; M = 4.734, SD = 1.303) than controls (M = 3.450, SD = 1.176), t(111) = 5.505, *p* < 0.001, Cohen’s d = 1.038 (95% CI [0.642, 1.429]). Conversely, the stroke group reported lower cognitive reappraisal (ERQ_Cog; M = 3.810, SD = 1.725) than controls (M = 5.727, SD = 1.309); due to unequal variances, the Welch test was used, t(96.358) = −6.587, *p* < 0.001, Cohen’s d = −1.263 (95% CI [−1.665, −0.855]; see Figure 3).

### 3.3 Emotion identification adjusted for general cognitive status

MoCA emerged as a significant between-subjects predictor of overall recognition accuracy (*F*(1, 110) = 29.125, *p* < 0.001, ηp^2^ = 0.209), indicating that higher cognitive screening scores were associated with higher identification accuracy across groups. The between-subjects effect of Group was not significant after controlling for MoCA (F(1, 110) = 1.501, *p* = 0.223, ηp^2^ = 0.014; adjusted means: stroke M = 2.921, controls M = 3.062), indicating that the overall accuracy difference between groups was substantially accounted for by MoCA differences. The main effect of Emotion was again robust (Greenhouse–Geisser: *F*(3.405, 374.55) = 64.314, *p* < 0.001, ηp^2^ = 0.369). Crucially, the Emotion × Group interaction retained significance in this model (*F*(5, 550) = 2.598, *p* = 0.025, ηp^2^ = 0.023; Greenhouse–Geisser: F(3.405, 374.55) = 2.598, *p* = 0.045, ηp^2^ = 0.023), demonstrating that the emotion-specific pattern of group differences was not fully accounted for by general cognitive status. The effect-size magnitude was identical to that observed in the unadjusted model (ηp^2^ = 0.023), indicating that MoCA control reduced the between-subjects overall accuracy difference without attenuating the within-subjects emotion-specific profile. The interaction only became non-significant in the fully extended model including both MoCA and ERQ indices as covariates (see Emotion identification adjusted for emotion regulation and global cognition, below).

### 3.4 Emotion regulation adjusted for general cognitive status

For expressive suppression (ERQ_Sup), the Group effect was significant after controlling for MoCA (F(1, 110) = 21.977, *p* < 0.001, ηp^2^ = 0.167; adjusted means: stroke M = 4.673, controls M = 3.504), whereas MoCA was not a significant predictor (F(1, 110) = 1.633, *p* = 0.204, ηp^2^ = 0.015). For cognitive reappraisal (ERQ_Cog), the Group effect was likewise significant (F(1, 110) = 33.906, *p* < 0.001, ηp^2^ = 0.236; adjusted means: stroke M = 3.881, controls M = 5.664), and MoCA was not a significant predictor (F(1, 110) = 1.484, *p* = 0.226, ηp^2^ = 0.013).

### 3.5 Emotion identification adjusted for emotion regulation

Box’s test indicated unequal covariance matrices across groups (Box’s M = 208.559, *p* < 0.001); therefore, multivariate results were interpreted primarily using Pillai’s trace. Sphericity was again violated (Mauchly’s W = 0.256, χ^2^(14) = 143.100, *p* < 0.001; εGG = 0.687), so Greenhouse–Geisser-corrected univariate tests were also examined.

The Emotion × Group interaction remained significant after adjustment (Pillai = 0.110, *F*(5, 103) = 2.545, *p* = 0.033, ηp^2^ = 0.110; Greenhouse–Geisser: *F*(3.437, 367.708) = 3.634, *p* = 0.010, ηp^2^ = 0.033), indicating that regulation differences did not account for the emotion-dependent stroke–control separation. In addition, suppression showed an emotion-dependent association with identification accuracy (Emotion × ERQ_Sup: Pillai = 0.128, F(5, 103) = 3.027, *p* = 0.014, ηp^2^ = 0.128; Greenhouse–Geisser: F(3.437, 367.708) = 3.234, *p* = 0.017, ηp^2^ = 0.029). In contrast, the Emotion × ERQ_Cog interaction was not significant (Pillai = 0.047, F(5, 103) = 1.017, *p* = 0.412; Greenhouse–Geisser *p* = 0.114). Higher-order interactions involving Group and ERQ were not significant at the multivariate level (Emotion × Group × ERQ_Cog: *p* = 0.143; Emotion × Group × ERQ_Sup: *p* = 0.078).

Bonferroni-adjusted simple effects (Group within each emotion), based on estimated marginal means evaluated at ERQ_Cog = 4.8276 and ERQ_Sup = 4.0522, showed significant group differences for anger (*Δ* = −0.518, SE = 0.183, *p* = 0.006) and disgust (Δ = −0.713, SE = 0.298, *p* = 0.018). Differences were not significant for joy (*p* = 0.429), sadness (*p* = 0.118), surprise (*p* = 0.675), or fear (Δ = −0.445, *p* = 0.102). Descriptively, the fear effect no longer reached the conventional significance threshold in the adjusted model, whereas the anger and disgust effects were maintained.

### 3.6 Emotion identification adjusted for emotion regulation and global cognition

Given that stroke survivors showed significantly lower MoCA scores than controls, and that subtle differences in general cognition might contribute to emotion recognition performance, a supplementary ANCOVA was estimated including MoCA as an additional covariate alongside ERQ_Cog and ERQ_Sup. Sphericity was again violated (Mauchly’s *W* = 0.276, χ^2^(14) = 136.531, *p* < 0.001; εGG = 0.681), so Greenhouse–Geisser corrections and Pillai’s trace are reported.

In this extended model, MoCA emerged as a robust predictor of recognition accuracy, both as a main effect (*F*(1, 108) = 30.157, *p* < 0.001, ηp^2^ = 0.218) and in interaction with Emotion (Pillai = 0.208, *F*(5, 104) = 5.476, *p* < 0.001; Greenhouse–Geisser: *F*(3.407, 367.936) = 7.187, *p* < 0.001), indicating that general cognitive status is differentially associated with recognition accuracy across emotion categories.

Critically, the Emotion × Group interaction was no longer significant after adjusting for MoCA (Pillai = 0.064, *F*(5, 104) = 1.421, *p* = 0.223; Greenhouse–Geisser: *F*(3.407, 367.936) = 0.737, *p* = 0.547), and the main effect of Group was attenuated to a trend (*F*(1, 108) = 2.759, *p* = 0.100). Bonferroni-adjusted simple effects showed no significant group differences for any individual emotion when covariates were held at their means (ERQ_Sup = 4.0522, ERQ_Cog = 4.8276, MoCA = 27.885), although anger retained a marginal trend (Δ = −0.309, *p* = 0.057).

Notably, the Emotion × ERQ_Sup interaction remained significant in this model (Pillai = 0.117, *F*(5, 104) = 2.758, *p* = 0.022; Greenhouse–Geisser: *F*(3.407, 367.936) = 2.664, *p* = 0.041), suggesting that the emotion-dependent association between expressive suppression and recognition accuracy is not reducible to general cognitive differences. In contrast, the Emotion × ERQ_Cog interaction remained non-significant (*p* = 0.136).

## 4 Discussion

The present study reveals a theoretically meaningful functional dissociation in the socioemotional sequelae of mild right-hemisphere ischemic stroke with excellent functional recovery (mRS < 2). In unadjusted models, stroke survivors showed lower overall facial emotion identification accuracy and a significant Emotion × Group interaction, with the clearest differences for anger, disgust, and fear. A sensitivity analysis including MoCA as the sole covariate clarified the structure of these effects: the between-subjects group difference in overall recognition accuracy was no longer significant when cognitive status was controlled, indicating that the global accuracy decrement was substantially tied to the mild cognitive sequelae accompanying right-hemisphere vascular injury. However, the Emotion × Group interaction retained significance in this model, with an identical effect size to the unadjusted model, indicating that the emotion-specific profile of group differences was not accounted for by cognitive status alone; the interaction only became non-significant in the extended model that simultaneously controlled for both MoCA and ERQ indices. By contrast, the stroke group showed large, robust differences in habitual emotion regulation —substantially higher expressive suppression and lower cognitive reappraisal— that were not explained by cognitive status: formal ANCOVAs confirmed that MoCA was not a significant predictor of either regulation strategy after accounting for group, and the emotion-dependent association between expressive suppression and recognition accuracy persisted after MoCA adjustment. Taken together, these findings indicate that whereas the overall level of emotion identification is sensitive to the mild cognitive sequelae of right-hemisphere stroke, habitual emotion regulation represents a cognitively independent dimension of socioemotional functioning that is robustly and independently affected by mild stroke, and it is at this level that the most persistent and theoretically distinctive post-stroke socioemotional mark is found.

An important methodological consideration concerns the precise scope of MoCA’s influence. Although both groups performed within the preserved range on the MoCA, stroke survivors scored significantly lower than controls (M = 27.15 vs. 28.53), and this difference has distinct implications for identification and regulation outcomes. For emotion identification, the sensitivity analysis with MoCA as the sole covariate demonstrated that general cognitive status accounted for the between-subjects group effect on overall accuracy but not for the Emotion × Group interaction, which retained its original effect size (the interaction). The interaction became non-significant only in the extended model that simultaneously controlled for both MoCA and ERQ indices, suggesting that cognitive and regulatory factors jointly —rather than separately— explain the emotion-specific group pattern. For emotion regulation, MoCA contributed negligibly and non-significantly to the prediction of either suppression or reappraisal, confirming that regulation differences reflect a dimension of post-stroke change that is independent of the mild cognitive sequelae captured by global screening. This dissociation —between cognitive sensitivity at the level of overall recognition accuracy and cognitive independence at the level of habitual regulation and the emotion-specific interaction pattern— is central to the interpretation of the functional dissociation reported in this study.

This pattern is also informative when interpreted through classic and contemporary accounts of emotional laterality (Blonder et al., 1991; Gainotti, 2024). The strongest and most persistent group differences were observed for anger and disgust, whereas fear showed a robust unadjusted group difference that was no longer statistically significant after accounting for regulation tendencies. This configuration is consistent with the hypothesis that right-hemisphere structures make differential contributions to the decoding of specific emotion categories, rather than a generic advantage for all nonverbal emotional communication. Critically, all six emotions tested here were conveyed through nonverbal, facial cues, making it impossible to attribute the selectivity of the impairment to a general deficit in processing nonverbal signals. Instead, the pattern points to category-specific dependencies on right-hemisphere nodes. Neuropsychological lesion studies and neuroimaging evidence indicate that the right anterior insula and adjacent opercular cortex are selectively implicated in the recognition and subjective experience of disgust; damage to these structures produces disproportionate disgust recognition impairment with relative sparing of other emotions (Calder et al., 2000; Adolphs, 2002). Similarly, the right amygdala and ventromedial prefrontal cortex are critically involved in processing facial signals of social threat, including anger, and lesions involving these right-hemisphere structures have been associated with selective anger recognition deficits (Adolphs, 2002; Tippett et al., 2018). Within this framework, the selective vulnerability of anger and disgust in our right-hemisphere stroke sample is grounded in the known functional architecture of these regions: both emotions depend heavily on right-hemisphere nodes specialized for threat- and aversion-related processing, making them disproportionately susceptible to disruption from right-hemisphere vascular lesions. The fact that fear also showed a group difference in the unadjusted model —but not after controlling for regulation— may reflect that fear recognition variance in this sample was partly shared with individual differences in expressive suppression, consistent with process-model accounts linking response-focused regulation to engagement with aversive signals (Gross, 1998; Gross and John, 2003). These mechanistic interpretations remain necessarily cautious, but they underscore that “good outcome” right-hemisphere stroke may selectively perturb the decoding of socially salient aversion- and threat-related emotions, while higher-level affective style may further modulate these effects across specific categories.

A further consideration speaks directly against the alternative interpretation that the observed group differences merely reflect differential task difficulty. If performance decrements in the stroke group were driven by a non-specific cognitive or perceptual decrement, one would expect group differences to concentrate systematically on the emotions that are intrinsically hardest to identify—those producing the lowest baseline accuracy in controls. However, this prediction is not borne out by the data. Anger was among the most accurately identified emotions in the entire sample: control group accuracy for anger was 3.73 out of 4 (93.3%), and stroke group accuracy was 3.34 (83.5%), placing anger well above the level of difficulty observed for disgust or fear. Yet anger also showed the most robust and consistent group difference across all models, surviving both the unadjusted ANOVA and the ERQ-adjusted ANCOVA with the highest effect size among individual emotions. This dissociation —between intrinsic task difficulty and magnitude of group difference— is precisely what a structure–function account would predict: anger recognition is specifically dependent on right amygdala and orbitofrontal circuits that are vulnerable to right-hemisphere vascular lesions, regardless of the overall ease of the emotion. A global cognitive difficulty account, by contrast, would predict that the easiest emotions should show the smallest group differences, which is the opposite of what is observed. This argument concerns specifically the emotion-category patterning of the group difference, not its overall magnitude. It is therefore complementary to, rather than contradicted by, the MoCA-adjusted analysis: the overall level of recognition accuracy is substantially tied to general cognitive status, but the emotion-specific profile —why anger, the easiest emotion in the task, shows the largest and most consistent group difference— is not predicted by any account based on uniform cognitive difficulty. Cognitive sequelae may thus explain how much stroke survivors struggle overall, while lesion-specific structural vulnerability explains which emotions are most affected.

Emotion-specific vulnerability after mild right-hemisphere stroke is the central behavioral implication of this work. The interaction between Emotion and Group indicates that stroke-related impairment is not a simple uniform decrement, but rather a change in the relative profile across emotions. This observation converges with broader evidence: social cognitive impairments are common after stroke (Adams et al., 2019) and emotion recognition deficits can be present even in individuals without overt global cognitive disorder (Aben et al., 2020). This adds a clinically relevant refinement: because all stroke participants had excellent functional recovery (mRS < 2) and MoCA scores in the non-impaired range, the observed alterations in the unadjusted model are not attributable to gross disability or frank global cognitive impairment, and the emotion-specific pattern provides clinically meaningful information about the socioemotional profile associated with mild right-hemisphere stroke. Nevertheless, as the MoCA-adjusted analysis demonstrates, emotion identification performance in this cohort is substantially tied to the level of general cognitive functioning: when cognitive status is equated across groups, the overall group accuracy difference largely disappears, although the emotion-specific interaction pattern is retained and only becomes non-significant in the extended model that simultaneously controls for both cognitive status and regulation indices. This finding does not diminish the clinical significance of socioemotional assessment in well-recovered patients; rather, it situates emotion identification deficits within the broader picture of mild cognitive sequelae that accompany right-hemisphere vascular injury, even when standard screening indicators remain within the preserved range.

A second contribution is the explicit bridge between facial emotion identification and habitual emotion regulation. In the process model, cognitive reappraisal and expressive suppression represent distinct regulatory routes with different downstream correlates, and reappraisal is typically associated with more adaptive affective and interpersonal profiles than suppression (Gross, 1998; Gross, 2014; Gross and John, 2003; Ochsner and Gross, 2005). The large group differences observed in the present study —higher suppression and lower reappraisal in the stroke group— suggest that mild right-hemisphere stroke may be associated with a shift in the style of emotional management, even when depressive symptom levels are low and comparable between groups. It is worth clarifying why depressive symptoms were not included as a covariate in the analyses of regulation: the relationship between regulation strategies and depressive symptomatology is not one of mere correlation, but of theoretical and empirical directionality. Habitual expressive suppression and reduced cognitive reappraisal are well-established antecedents and maintaining factors of depressive symptoms in non-clinical populations —they predict prospectively lower positive affect, higher negative affect, and reduced wellbeing (Gross and John, 2003)— rather than consequences of depression. Adjusting for BDI in models where the dependent variables are the ERQ indices would therefore risk removing precisely the variance most meaningful for characterizing the post-stroke regulatory profile, constituting a form of overadjustment bias. Although causal direction cannot be inferred from the present cross-sectional data, this pattern is consistent with the possibility that subtle changes in socioemotional processing following stroke may promote reliance on inhibition of emotional expression, while reducing the accessibility or habitual use of reinterpretation-based strategies. Importantly, these regulation differences were not merely epiphenomenal: they were sufficiently pronounced to warrant being treated as a core feature of the post-stroke profile, not simply an ancillary measure.

At the same time, the analyses indicate that regulation does not offer a simple explanation for the recognition deficits. The Emotion × Group interaction remained significant when ERQ indices were included as covariates, and the clearest between-group differences persisted for anger and disgust. This argues against an account in which altered recognition is simply “carried” by group differences in regulation tendencies.

The most nuanced integration emerges from the ANCOVA: expressive suppression showed an emotion-dependent association with recognition accuracy, whereas reappraisal did not. This is theoretically interesting because suppression is classically conceptualized as a response-focused strategy —one that modifies expression after emotion is generated (Gross, 1998)— yet it appears to covary with accuracy in an emotion-contingent manner. One parsimonious interpretation is that suppression functions here as a marker of a broader socioemotional stance (e.g., diminished engagement with affective cues, interpersonal reticence, or reduced expressive feedback) that selectively affects decoding of certain facial signals. This interpretation is further supported by the well-documented association between right-hemisphere stroke and reduced facial expressiveness: stroke survivors with right-hemisphere lesions frequently show diminished spontaneous facial affect expression (Borod et al., 1986; Blonder et al., 1993), and it is plausible that habitual suppression of expressive output and reduced sensitivity to incoming facial signals may form a functionally coherent profile within this group, each reinforcing the other. Another interpretation is that suppression overlaps with stable differences in attentional allocation or aversive engagement that influence recognition especially for threat-related categories, which would align with the pattern in which the fear effect was no longer significant after covariate adjustment. These interpretations are compatible with accounts emphasizing that emotional meaning is shaped by both perceptual signals and higher-level processes that modulate attention and interpretation (Barrett, 2022; Ochsner and Gross, 2005). In this sense, these findings provide an empirically grounded example of how a “complex” dimension of emotional functioning (habitual regulation style) can relate to performance in a task often considered “basic” (categorical identification), but in a selective manner that becomes visible only when emotion-specific structure is preserved.

A theoretically important question raised by the elevated expressive suppression observed in the stroke group concerns the interpretation of this self-reported regulatory tendency in the context of the well-established literature on reduced emotional expressivity following right-hemisphere damage. Several decades of research have documented that patients with right-hemisphere lesions show markedly reduced facial expressivity compared to both left-hemisphere-damaged patients and neurologically healthy controls, both in laboratory settings and during spontaneous conversation (Borod et al., 1986; Blonder et al., 1993). This pattern —often described in terms of flat affect, hypomimia, or diminished affective output— has been replicated across facial, prosodic, and lexical channels of emotional communication (Borod et al., 1998), and is considered a core, though frequently underassessed, sequela of right-hemisphere stroke (Erhan et al., 2000; Cummings, 1997). Crucially, right-hemisphere damage has also been associated with higher rates of alexithymia —a construct characterized by difficulties in identifying and describing emotional states— which has itself been linked to a regulatory profile dominated by expressive suppression and lower use of cognitive reappraisal (Preece et al., 2023; Swart et al., 2009). Against this background, the elevated ERQ suppression scores observed in our stroke group admit of at least two non-mutually exclusive interpretations. First, they may reflect a volitional regulatory shift: following stroke, survivors may deliberately inhibit emotional expression as an adaptive interpersonal strategy, particularly in contexts where reduced ability to read others’ emotions increases social uncertainty and promotes a defensive, inhibitory stance toward affective communication. Second, and perhaps more parsimoniously, elevated self-reported suppression may partially capture the neurologically driven reduction in expressive output that is characteristic of right-hemisphere damage—that is, patients may retrospectively attribute their reduced expressivity to a deliberate regulatory strategy when it is in fact, at least in part, a direct consequence of lesion-related disruption of the right-hemisphere networks that mediate spontaneous emotional expression (Borod et al., 1986; Blonder et al., 1993). These two accounts are not easily separable using self-report measures alone, and their relative contributions cannot be disentangled in the present cross-sectional design. Future work incorporating behavioral measures of spontaneous expressivity alongside self-report regulation indices would allow a more precise characterization of whether the suppression profile observed here reflects regulatory agency, neurological constraint, or their interaction.

Clinically, the results support the importance of extending post-stroke characterization beyond traditional endpoints. Patients with minimal disability may nonetheless experience subtle socioemotional alterations that can shape interpersonal functioning and participation. From an applied perspective, two implications follow. First, socioemotional screening could be considered even in “well-recovered” patients, particularly when reintegration into complex social environments is a priority. Second, the large group differences in regulation style raise the possibility that post-stroke support might benefit from addressing regulation strategies explicitly, not only mood symptoms. Even if regulation does not cause recognition impairment, both domains may interact in daily life: difficulties in reading others’ emotions could encourage suppression as a protective interpersonal strategy, while elevated suppression could reduce opportunities for corrective social feedback and emotional learning.

Several limitations should be considered. The cross-sectional design precludes determining whether regulation differences predated the stroke or emerged during recovery. The control group scored statistically higher on MoCA, and the MoCA-adjusted analyses demonstrate that this difference substantially accounts for the between-subjects group effect on overall emotion identification accuracy, although the Emotion × Group interaction retained significance when MoCA was the sole covariate and only became non-significant in the extended model jointly controlling for both MoCA and ERQ indices; future work should incorporate more detailed executive function measures to better characterize the specific cognitive dimensions that covary with socioemotional performance. Manual response recording and unlimited response time maximized accuracy validity (García-Rodríguez et al., 2009, 2012a, 2012b) but prevented analysis of processing speed, decisional thresholds, or speed–accuracy tradeoffs; adding response-time and/or signal-detection approaches could clarify whether deficits reflect perceptual sensitivity, category boundary uncertainty, or decisional conservatism (Macmillan and Creelman, 2005). Regulation was measured by self-report (Gross and John, 2003; Cabello et al., 2013), which captures habitual tendencies but may be influenced by insight and social desirability (Mauss and Robinson, 2009); multimethod assessment (behavioral tasks, informant ratings, ecological momentary assessment) would strengthen inference. While avatar-based stimuli improve standardization and have validation support (Moser et al., 2007), generalization to dynamic, naturalistic, multimodal emotional communication warrants direct testing. The four-item-per-emotion structure of the EFE task produces relatively imprecise per-category accuracy estimates compared to instruments with higher item density (e.g., the Ekman 60 Faces Test); this may have attenuated the detectability of emotion-specific effects and should be addressed in future replications. Furthermore, only right-hemisphere stroke survivors were examined, which precludes direct causal attribution of the observed deficits to hemispheric specificity. Available evidence suggests that emotion recognition difficulties are not invariably restricted to right-hemisphere damage (Aben et al., 2020; van den Berg et al., 2021), and future work including left-hemisphere stroke comparison groups would be essential to determine the laterality specificity of the present findings. Finally, the absence of neuroimaging data with sufficient resolution for formal lesion-symptom mapping is a further constraint: linking the observed emotion-specific profiles to lesion location and network connectivity metrics would substantially advance the mechanistic interpretation of the results (van den Berg et al., 2021).

Future work could extend this line in several directions. One is to introduce context manipulations —situational frames, social cues, or complex emotions that require appraisal and language— to directly address how context shapes emotional meaning and to connect more explicitly with constructionist perspectives (Barrett, 2022). Another is to develop the “how do they do it?” analysis by formally modeling confusion patterns (e.g., fear-as-surprise confusions) and examining whether suppression predicts specific misclassifications, which would provide a process-level account beyond mean accuracy. Finally, intervention-oriented studies could test whether training in emotion recognition and/or regulation strategies improves social participation outcomes, emphasizing socioemotional functioning as a legitimate component of recovery rather than a residual afterthought.

Taken together, the present findings invite a reconceptualization of what “good recovery” from mild stroke entails. Standard endpoints —functional independence and global cognitive screening in the normal range— are insufficient to characterize the full scope of post-stroke adaptation. Socioemotional processing, including the ability to decode others’ emotional expressions and the regulatory strategies deployed in daily life, appears to constitute a distinct dimension of outcome that can be altered even when conventional indicators are reassuring. Understanding the neural and psychological mechanisms underlying these alterations, and testing whether they are amenable to targeted intervention, represents a clinically meaningful research agenda at the interface of cognitive neuroscience and stroke rehabilitation.

## 5 Conclusion

Overall, the findings support the inclusion of dedicated socioemotional assessment —and particularly the characterization of habitual emotion regulation strategies— in the clinical follow-up of well-recovered stroke survivors. Conventional neurological recovery metrics do not capture the socioemotional dimensions that may remain altered even when standard endpoints are reassuring. Future studies should link these profiles to lesion and network markers, examine how regulation-dominant suppression profiles contribute to social participation outcomes, and test whether targeted emotion regulation interventions can improve quality of life and social reintegration after mild stroke.

## Data Availability

The raw data supporting the conclusions of this article will be made available by the authors, without undue reservation.
